# Ketogenic diet and BHB rescue the fall of long-term potentiation in an Alzheimer’s mouse model and stimulates synaptic plasticity pathway enzymes

**DOI:** 10.1038/s42003-024-05860-z

**Published:** 2024-02-16

**Authors:** Jacopo Di Lucente, Giuseppe Persico, Zeyu Zhou, Lee-Way Jin, Jon J. Ramsey, Jennifer M. Rutkowsky, Claire M. Montgomery, Alexey Tomilov, Kyoungmi Kim, Marco Giorgio, Izumi Maezawa, Gino A. Cortopassi

**Affiliations:** 1 Department of Pathology and MIND Institute, University of California Davis Medical Center, Sacramento, CA 95817 USA; 2 Department of Experimental Oncology, European Institute of Oncology, IRCCS, 21041 Milan, Italy; 3 Department of Molecular Biosciences, School of Veterinary Medicine, University of California Davis, Davis, CA 95616 USA; 4 Alzheimer’s Disease Research Center, University of California Davis Medical Center, Sacramento, CA 95817 USA; 5 Department of Public Health Sciences, School of Medicine, University of California Davis, Davis, CA 95616 USA; 6 Department of Biomedical Sciences, University of Padova, 35131 Padova, Italy

**Keywords:** Alzheimer's disease, Fat metabolism

## Abstract

The Ketogenic Diet (KD) improves memory and longevity in aged C57BL/6 mice. We tested 7 months KD vs. control diet (CD) in the mouse Alzheimer’s Disease (AD) model APP/PS1. KD significantly rescued Long-Term-Potentiation (LTP) to wild-type levels, not by changing Amyloid-β (Aβ) levels. KD’s ‘main actor’ is thought to be Beta-Hydroxy-butyrate (BHB) whose levels rose significantly in KD vs. CD mice, and BHB itself significantly rescued LTP in APP/PS1 hippocampi. KD’s 6 most significant pathways induced in brains by RNAseq all related to Synaptic Plasticity. KD induced significant increases in synaptic plasticity enzymes p-ERK and p-CREB in both sexes, and of brain-derived neurotrophic factor (BDNF) in APP/PS1 females. We suggest KD rescues LTP through BHB’s enhancement of synaptic plasticity. LTP falls in Mild-Cognitive Impairment (MCI) of human AD. KD and BHB, because they are an approved diet and supplement respectively, may be most therapeutically and translationally relevant to the MCI phase of Alzheimer’s Disease.

## Abstract (web summary)

A Ketogenic Diet (KD) that improves memory in aged C57BL/6 mice is tested in APP/PS1 Alzheimer’s mice. Seven months KD rescues Long-Term-Potentiation to wild-type levels, as does a one-hour soak with 3 mM Betahydroxybutyrate. KD increases synaptic pathways but does not alter Aβ amyloid.

## Introduction

Alzheimer’s disease is a progressive neurodegenerative disorder characterized by cognitive deficits and synaptic dysfunction. The hallmarks of AD include the cleavage of the amyloid precursor protein (APP) by secretases and the accumulation of amyloid peptides (Aβ)^1^ in the extracellular space. Aβ molecules can aggregate to form soluble oligomers (AβO) that can accumulate into insoluble plaques and that these ultimately result in memory loss and neurodegeneration^2,3^. A more recent extension of AD theory is that Aβ may exert its primary neurotoxicity at the synapse.

Long-term potentiation (LTP) is a neurophysiological measurement related to synapse-based learning and memory^4^. During LTP, some stronger synaptic connections are reinforced, and weaker ones are removed, resulting in the refinement of synaptic connections. LTP is reduced in animal models of AD that overexpress Aβ and experience memory loss, consistent with the idea that an Aβ dependent synaptic deficit may be an early event in AD pathophysiology^5–8^.

The main pathway thought to underlie LTP synaptogenesis is a protein phosphorylation cascade involving cAMP-element binding protein (CREB), calcium/calmodulin-dependent protein kinase II (CaMKII), and extracellular signal-regulated kinase^9–14^.

The ketogenic diet (KD) is a low-carbohydrate, high-fat, and moderate protein diet, that was demonstrated by two groups to increase memory, cognition and longevity even when introduced at 12 months of age in C57BL/6 mice^15,16^. The ketone body β-hydroxybutyrate (BHB) is thought to be the KD’s main ‘actor’. BHB rises in the fasting state with lipids provided by peripheral adipocytes and rises to a higher level in KD vs. CD diets^15,16^. BHB penetrates the blood-brain barrier and signals downstream pathways through its hydroxycarboxylic acid receptor (HCAR2) and can also be used as a neural energy source^17,18^.

KD has been used since 1921 to suppress epileptic seizures in humans and is still the therapy of choice for juvenile recurrent seizures, although the mechanism is unknown^19^. The KD’s efficacy has been supported in human cognitive impairment^20^, neurological disorders and inflammation^21–23^

In this study, we scrutinized the impact of the KD on synaptic plasticity in the AD mouse model APP/PS1. We show 7 months of KD rescues deficient LTP in APP/PS1 mice. RNAseq of KD vs. CD mice supports the concept that KD increases synaptic plasticity. Biochemical studies of KD hippocampi support the idea that KD increases enzymes involved in synaptic plasticity, and KD didn’t alter hippocampal Aβ levels. BHB levels significantly rose in the KD mice. We propose that KD rescues impaired LTP in APP/PS1 mice not through a reduction of Aβ but through activating synaptic signaling cascade, and a model for this is presented.

## Results

### Overall, the study design and 7 months of ketogenic diet do not affect body composition in APP/PS1

We and others showed a ketogenic diet (KD) intervention for 12–13 months improved cognition memory, and longevity relative to the standard carbohydrate-rich diet in aged wild-type C57BL/6 mice^15,16^. In the current study, we tested whether a 7-month intervention of KD initiated at month 6 could affect LTP deficits that are normally observed at month 8 and beyond in APP/PS1 mice consuming CD^24^. The overall study design is shown in Supplementary Fig. 1. Body weight, lean mass, fat mass, and percent body fat were not affected by KD (Supplementary Fig. 2a–d).

### KD and its active principle BHB rescue the APP/PS1-dependent LTP deficit observed on CD

**Fig. 1:**
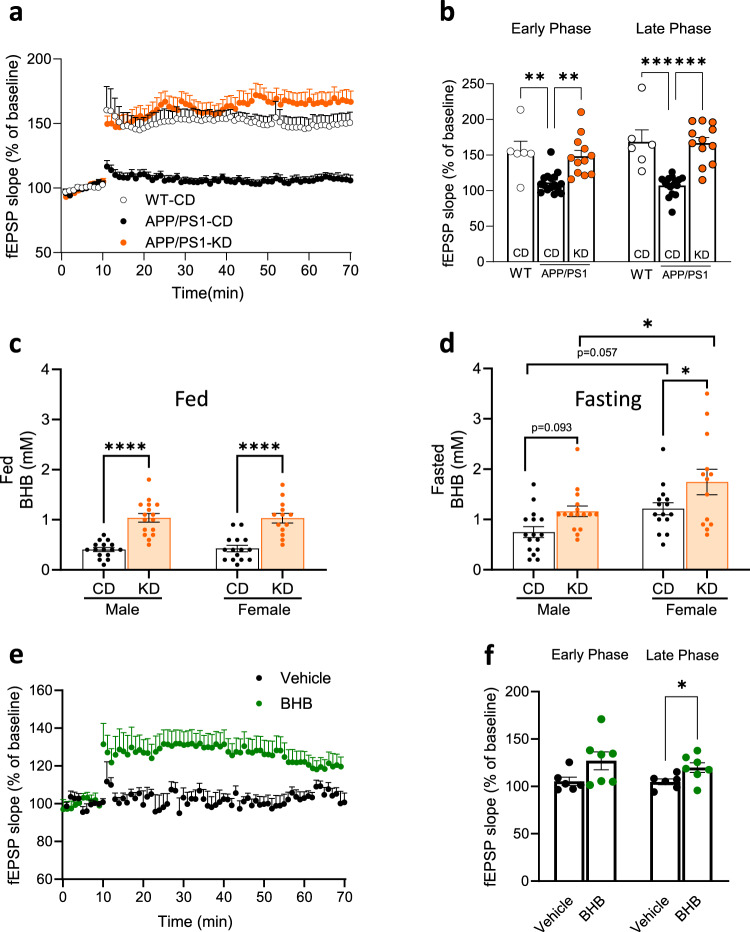
Ketogenic diet and BHB prevent LTP impairments in APP/PS1 mice. **a** HFS-induced LTP was impaired in slices from 13-month-old male (*n* = 4) and female (*n* = 4) APP/PS1 mice, compared with slices from wild-type mice (*n* = 6). In KD-treated APP/PS1 slices, HFS included LTP similar to wild-type (*n* = 3 male and 3 female). **b** Cumulative data showing mean fEPSP slopes 3 min (early phase) or 50–60 min (late phase) post-HFS. **c** Blood β-hydroxybutyrate was significantly increased in both males and females in the KD versus CD groups in the fed state (measured 3 h after feeding). Two-way ANOVA with Tukey’s post-hoc tests shows significant differences between diet treatments (*F*(1,56) = 70.96, *p* < 0.001). **d** Blood β-hydroxybutyrate levels were significantly increased in female mice in the KD versus CD groups following a 12-h overnight fast (*n* = 13–16 per group). Two-way ANOVA with Tukey’s post-hoc tests shows significant differences between diet treatments (*F*(1,56) = 10.14, *p* = 0.0024) and between genders (*F*(1,56) = 12.44, *p* = 0.0008). **e** BHB-treat**e**d APP/PS1 slices show increased LTP compared to vehicle-treated APP/PS1 slice (*n* = 2 male and 2 female). **f** Cumulative data showing mean fEPSP slopes 3 min (early phase) or 50–60 min (late phase) post-HFS. Data are presented as median ± interquartile range. **p < 0.01, ***p < 0.001, Kruskal–Wallis statistic = 15.51 and *p* < 0.001 for early phase. Kruskal–Wallis statistic = 22.68 and *p* < 0.001 for the late phase. Data for BHB experiments are presented as mean ± SEM *p < 0.05, unpaired *t*-test.

To explore the effect of KD on Aβ-related impairments in hippocampal synaptic plasticity, we used slices from APP/PS1 mice. Hippocampal LTP is the foundation of memory and of synaptic plasticity^25^. Hippocampal LTP in 13-month CD-fed APP/PS1 mice was deficient, compared with wild-type animals (Fig. 1a, b) after high-frequency stimulation in the CA3–CA1 pathway, consistent with our previously described learning and memory deficits in these mice^26^. By contrast, 7 months of the ketogenic diet preserved both early and late-phase LTP to within wild-type 13-month C57BL/6 LTP levels (Fig. 1a, b).

### Blood BHB levels are increased in APP/PS1 mice after the ketogenic diet

Beta-hydroxy Butyrate, BHB, is considered to be an ‘active principle’ of the KD^16^. To test this idea, BHB was measured in mouse blood at 3 h after meal feeding (fed state, Fig. 1c) and after a 12 h overnight fast (Fig. 1d) in the APP/PS1 mice. In the fed state, circulating BHB levels were significantly increased in KD versus CD groups for both male and female mice (Fig. 1c). In the fasted state, mean blood BHB levels were higher than in the fed state (Fig. 1c vs. Fig. 1d), about 1.2–1.6 mM. The fasting BHB levels were significantly higher in females KD vs. CD (*p* = 0.04), but only trended in that direction in males (*p* = 0.093).

### A 1-h soak of 3 mM BHB provides significant LTP rescue in APP/PS1 mice

Since BHB is a major active ketone body following KD consumption, we assessed the effect of BHB on LTP by bath-incubating brain slices from 15-month-old APP/PS1 mice with BHB. LTP deficit in APP/PS1 was rescued by 1 h pre-incubation with BHB, which significantly elevated the maintenance/late phase (Fig. 1e, f) although the slope of fEPSP in BHB-incubated slices was slightly less than the slope observed in KD-treated mice. This result suggests that BHB alone is sufficient to recover beta amyloid-induced LTP deficits.

### Gene ontology supports the view that KD alters synaptic plasticity in APP/PS1  mice

**Fig. 2:**
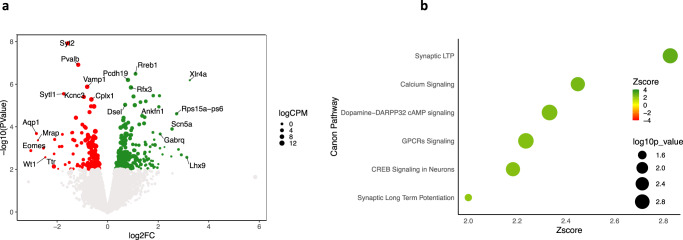
Gene Ontology supports the view that KD alters synaptic plasticity in APP/PS1 mice. **a** Volcano plot of differentially expressed genes (DEGs) induced by KD. Upregulated genes are shown in green, while downregulated genes are shown in red. Genes with log2FC greater than 2 or lower than −2 or the most significant are also labeled. The dimension of dots reflects the logCPM (logarithm of counts per million reads). **b** Ingenuity Pathway Analysis of genes influenced by diet. Canonical pathways with absolute *z*-score greater than 2 and −log *p*-value > 1.3 are reported in green, while dimensions of dots reflect FDR values as indicated in figure.

RNA-seq was conducted using four biological replicates for the control group (CD) and three replicates for KD treated group. After the removal of the lowest expressed genes, differential analysis by edgeR package allowed us to identify genes affected by diet. We found 354 differentially expressed genes (DEGs, (*p*-value ≤ 0.01)), 141 downregulated and 213 upregulated genes as reported in Fig. 2a (volcano plot) colored respectively in red and green while dots size is relative to logCPM (logarithm of counts per million reads) of the gene. Differentially expressed genes are then used as input for Gene Ontology Analysis using Ingenuity Pathway Analysis; all significant canonical pathways (*z*-score > 2 and −log *p*-value > 1.3) are plotted in Fig. 2b. As shown in Fig. 2b, of the 6 significant induced pathways all 6 related to synaptic plasticity, including Synaptic Long-term depression, CREB signaling in neuron, synaptic long-term potentiation, GPCR signaling, calcium signaling, and dopamine-DARPP32 cAMP signaling, supporting the view that KD has a significant impact on synaptic plasticity.

### The KD significantly activates enzymes involved in synaptic plasticity in both male and female mice

**Fig. 3:**
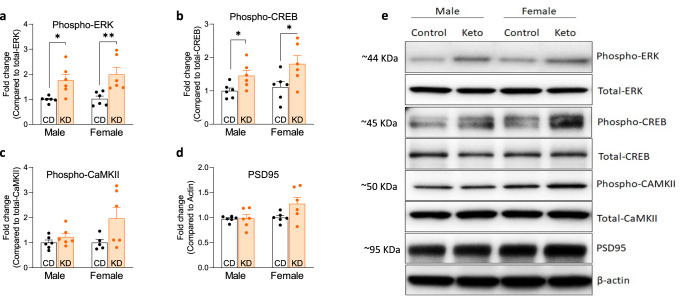
Ketogenic diet activates CREB and ERK but not CaMKII in the hippocampus of APP/PS1 mice. **a**–**d** Relative protein level of phospho-CREB, phospho-ERK, phospho-CaMKII, and PSD95. Six-month treatment with a ketogenic diet increases the activation of CREB and ERK in APP/PS1 mice but does not affect CaMKII or PSD95 (*n* = 6). **e** Representative immunoblot of phosphorylated and total proteins. Data are presented as mean ± SEM. *p < 0.05, **p < 0.01. Two-way ANOVA tests show significant differences between diet treatments in phospho-ERK: *F*(1,20) = 19.97, *p* < 0.001; phospho-CREB: *F*(1,20) = 10.84, *p* = 0.004; phospho-CamKII: *F*(1,19) = 5.344, *p* = 0.032.

It is known that depolarization and activation of NMDA receptor by presynaptic released glutamate allows Ca^2+^ entry into dendritic spines, which triggers the activation of cell signaling pathways such as Ras–extracellular signal-regulated kinase^9^ and Calcium/ calmodulin-dependent kinase II (CaMKII) that eventually lead to gene transcription and protein synthesis required for learning and memory^27^. To investigate whether KD can activate those protein kinases crucial for LTP^28,29^, we scrutinized ERK and CREB activity in the hippocampus. Phospho-ERK and its downstream transcription factor CREB were significantly elevated in hippocampi of KD-fed APP/PS1 male and female mice compared to CD-fed APP/PS1 mice; their total protein levels were not changed, consistent with the idea of pathway activation (Fig. 3a, b, e). Since ERK and CREB play an important role in consolidating short-term memory to long-term memory^27^, our result suggests that KD/BHB increases p-ERK activation, which supports early-phase LTP, and p-CREB activation, which supports late-phase LTP.

### KD does not appear to rescue LTP by reducing Aβ amyloid burden

Another possible mechanism of KD’s rescue of LTP by KD is the reduction of Aβ amyloid burden^28,30,31^. Using ELISA to quantify Aβ extracted from the brain, we found no significant differences between KD- and CD-fed APP/PS1 mice in Aβ species in both TBS-soluble and TBS-insoluble/SDS-soluble fractions (Supl. Fig. 3).

### KD significantly reduces markers of microgliosis but not astrogliosis

**Fig. 4:**
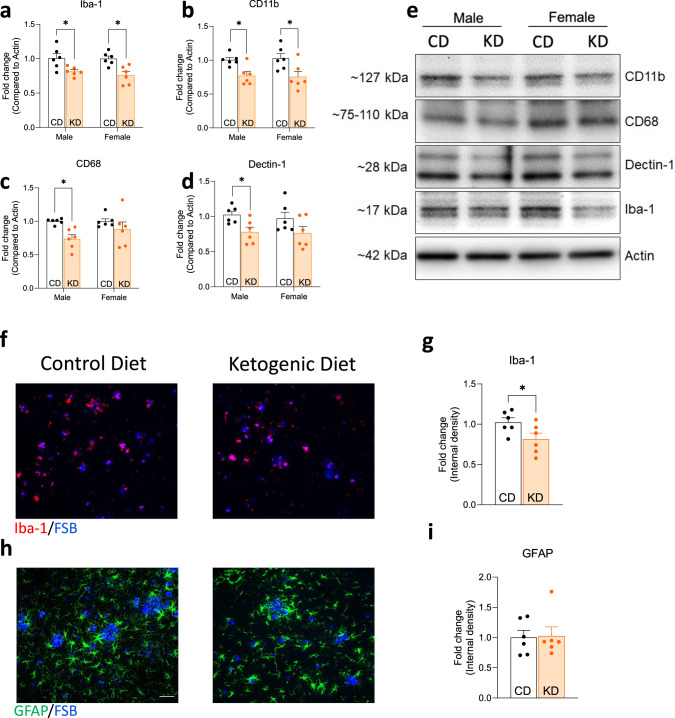
Ketogenic diet reduces microglial activation in APP/PS1 mice. **a**–**d** Relative protein level of CD11b, CD68, Dectin-1, and Iba-1 (*n* = 6). **e** Representative immunoblot of microglial activation proteins. **h** Representative images of astrocytes labeled by GFAP immunostaining in the CA1 area of the hippocampus of APP/PS1 mice. **i** Quantification of GFAP internal density (*n* = 3 male and 3 female). **f** Representative images of microglia labeled by Iba1 immunostaining in the CA1 area of the hippocampus of APP/PS1 mice. **g** Quantification of Iba1 internal density (*n* = 3 male and 3 female). Data are presented as mean ± SEM *p < 0.05, **p < 0.01, *n* = 6. Two-way ANOVA tests show significant differences between diet treatment in Iba-1 *F*(1,20) = 16.60, *p* < 0.001; CD11b *F*(1,20) = 11.64, *p* = 0.003; CD68 *F*(1,20) = 8.563, *p* = 0.008; and Dectin-1 *F*(1,20) = 7.619, *p* = 0.012. Data for immunostaining experiments are presented as mean ± SEM *p < 0.05, unpaired *t*-test.

Previous studies showed microglial and astrocyte hyperactivation play a pivotal role in eliciting inflammation in AD brain^32^. APP/PS1 mice develop gliosis around 4 months of age, which continues to later age^33^. Thus, we assessed KD/BHB’s effects on gliosis. Microglial markers Iba1 and CD11b were significantly reduced by KD in both male and female APP/PS1 mice (Fig. 4a, b, e). CD68, a microglia/macrophage maker, and Dectin-1, a disease-associated microglia marker, were significantly reduced in male but not female APP/PS1 mice by KD (Fig. 4c–e). Immunofluorescence staining further confirmed the reduction of Iba1-reactive microglia by KD (Fig. 4f, g). In contrast to multiple significant reductions in biomarkers of microgliosis, there was no change in a biomarker of astrogliosis, i.e., GFAP^-^reactive astrocytes between CD- and KD-fed APP/PS1 mice (Fig. 4h, i). Taken together, the results of the previous two paragraphs support the view that KD/BHB may rescue LTP in APP/PS1 mice by reducing microglial inflammation without affecting Aβ deposits.

### A ketogenic diet increases BDNF levels in females but not males

**Fig. 5:**
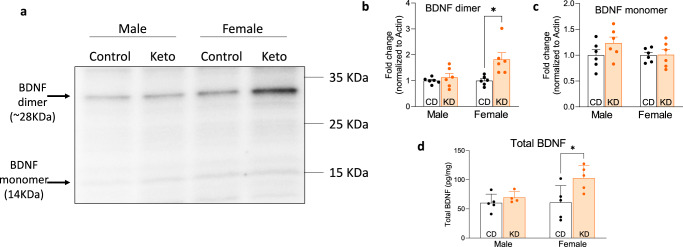
Ketogenic diet increases BDNF level in the hippocampus of female APP/PS1. **a** Representative immunoblot and quantitative analysis (**b**, **c**) of BDNF dimer and monomer in the hippocampus of APP/PS1 mice. KD-fed female mice show increased BDNF dimer. *n* = 6. **d** Hippocampal BDNF level in APP/PS1 mice detected by ELISA. KD-fed female mice show increased levels of brain-derived neurotrophic factor compared to CD-fed littermates. *n* = 5. Data are presented as mean ± SEM. *p < 0.05. Two-way ANOVA tests show significant differences between diet treatments (*F*(1,20) = 9.156, *p* = 0.007) and gender differences (*F*(1,20) = 4.914, *p* = 0.038) in BDNF dimer. The analysis also shows significant gender differences in total BDNF (*F*(1,16) = 4.540, *p* = 0.049).

BDNF supports LTP and lies downstream of p-ERK and p-CREB^34^, which were significantly altered by KD (Fig. 3a, b). BDNF enhances glutamate release in synaptosomes^35^ that eventually activates AMPA/NMDA receptors to support LTP. Mattson’s group previously showed that BHB enhances BDNF production in cultured hippocampal neurons^36^. Thus, we investigated BDNF expression in KD-fed APP/PS1 mice (Fig. 5). There are two forms of BDNF, 14 kDa monomer and 28 kDa dimer^37^. Western blot analysis of hippocampal homogenates detected both BDNF-monomer and -dimer (Fig. 5a–c), and the only significant difference between groups was increased level of BDNF-dimer in KD-fed female APP/PS1 mice compared to CD-fed female APP/PS1 mice (Fig. 5b). This result is further confirmed by ELISA quantification of BDNF, which employed a different set of antibodies (Fig. 5d). This suggests that while upstream LTP supporting factors p-ERK and p-CREB are significantly activated by KD in hippocampi of both sexes, that downstream BDNF only significantly rises in females, we attribute this to the significantly higher fasting BHB levels in females than males, see Fig. 1d and “Discussion”.

### A ketogenic diet increases exploration in APP/PS1 mice, with a potential impact on working memory

LTP decline is the earliest neurophysiologically measurable feature in both APP/PS1 mice^24^ and in human AD^38,39^. Other deficits occur later; we measured locomotor activity, working memory, and spatial learning memory in APP/PS1 mice using the open field, Y-maze, and Barnes maze tests. In the open field test, females explored and covered more distance than males (Supl. Fig. 4a, sex *p* < 0.001), and animals on a ketogenic had greater spontaneous movement as indicated by a further distance traveled during the test than mice fed a control diet (*p* < 0.05). This increased movement/exploration by APP/PS1 female mice and APP/PS1 mice fed a KD was also seen in the Y-maze test (*p* < 0.01) with a strong trend for a sex × diet interaction (Supl. Fig. 4b, interaction *p* = 0.051). In the Y-maze test, there was also a strong trend for animals on a KD to have improved working memory, as indicated by an increase in the percentage of alternating triplets when compared to control-fed animals (Supl. Fig. 5a, *p* = 0.0610). No difference in spatial learning memory by diet was observed as assessed by the Barnes maze (Supl. Fig. 5b, c latency and % time in target quadrant). There was a sex effect in the Barnes maze test where females showed decreased spatial memory as indicated by decreased % time in target quadrant compared to male mice (Supl. Fig. 5c, *p* < 0.01). So, while the KD and BHB itself have significant effects on LTP, synaptic plasticity enzymes p-ERK and p-CREB, and BDNF expression in females that relate to the early phase of AD known as mild cognitive impairment (MCI), their impact was not as strong on what could be considered the later and more integrative phases of the disease.

## Discussion

We and others showed a ketogenic diet (KD) intervention for 14 months improved cognition memory, and longevity relative to the standard carbohydrate-rich diet (CD) in aged wild-type C57BL6 mice^15,16^. One interpretation of those results is that KD ‘slows aging’, and thus in accord with the ‘geroscience’ concept, the KD could potentially slow other age-related diseases such as Alzheimer’s^40^. We tested KD in the APP/PS1 mouse, which overexpresses mutant human APP, and Presenilin, which has been tested in hundreds of research publications as a model of human AD pathophysiology.

We observed that KD significantly rescued both early and late-phase hippocampal LTP in APP/PS1 animals to within wild-type C57BL/6 control levels (Fig.1a, b). The KD’s “driver” is thought to be BHB, whose level rises on KD Fig. 1c, d orange bars. We tested the benefit of BHB incubation for one hour of hippocampal slices from APP/PS1 mice and observed a significant rescue. These data support the view that most of KD’s neurophysiological LTP benefit, driven by eating the KD for 7 months, can be approximated by a one-hour incubation of hippocampal slices with BHB ex vivo. This suggests BHB is a pharmacological means to support LTP in AD.

Our earliest thought was that KD might provide synaptic LTP benefit through the reduction of Aβ itself, but the lack of any effect of KD on Aβ (Supl. Fig. 3) or body composition (Supl. Fig. 2) suggested there must be other mechanisms for KD’s rescue of LTP, and we scrutinized the known biochemical pathways for synaptic plasticity ERK → CREB → BDNF, Gene Ontogeny pathways by RNAseq, and the potential involvement of microglial or astroglial activation and inflammation.

RNAseq analysis of APP/PS1 cortices on KD vs. CD demonstrated many genes significantly altered (Fig. 2a). Processing these by the Gene Ontology program Ingenuity Pathway Analysis (IPA) identified 6 significantly upregulated pathways all related to synaptic plasticity, suggesting that KD’s mechanism of action to rescue LTP is through improved synaptic plasticity (Fig. 2b).

**Fig. 6:**
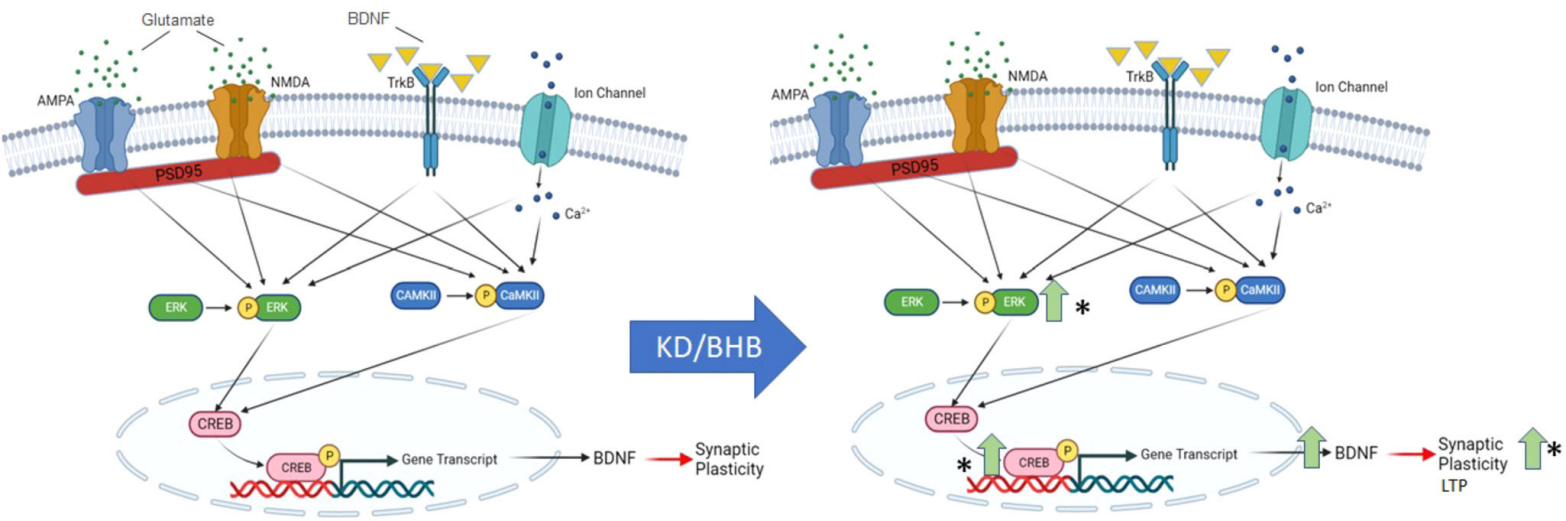
A biochemical model of pathways supporting Synaptic Plasticity. Glutamatergic receptors (AMPA and NMDA), intracellular Ca^2+^, and TrkB activity stimulate the phosphorylation of ERK and CaMKII, which subsequently enhance the phosphorylation of CREB. Activation of CREB regulates gene expression necessary for the formation of long-term memory, including BDNF. A ketogenic diet increases the phosphorylation of the ERK/CREB pathway, increasing BDNF production and synaptic plasticity.

We then investigated biochemical pathways thought to underlie synaptic plasticity in the hippocampi of KD and CD-fed mice (Fig. 6)^41^. KD increased LTP in male and female mice (Fig. 1), and KD significantly increased phospho-ERK and phospho-CREB pathways in both sexes’ hippocampi that are proposed to support synaptic plasticity (Fig. 3a, b)^42^. Thus, we interpret that KD’s pro-synaptogenic effects in APP/PS1 are more likely to be elicited through a mechanism such as KD > TrkB > p-ERK > p-CREB > LTP (i.e., the pathway on the left side of Fig. 6), than the pathway KD > PSD95 > CaMKII > LTP for which we observe no significant support in Fig. 3c, d. We are currently studying the consequences of a ‘KetoDrug’/Shc inhibitor that may engage downstream of TrkB that could engage a similar mechanism as KD.

By contrast to the sex-independent effects of KD on p-ERK and p-CREB synaptic pathways, KD’s effect on one downstream consequence, BDNF, appears much more sex-specific, i.e., significant in females but not in males (Fig. 5a–c). One possible interpretation is sex-specific differences in synaptic signaling cascades impacting learning and memory^27,43^. Another possible explanation is sex-specific differences in BHB levels, as described in the paragraph below. Mattson’s group has shown that BHB enhances BDNF production in cultured hippocampal neurons^36^, so if BHB levels were higher in females, this could potentially explain the BDNF difference. BHB rises in both fed and fasting phases on KD vs. CD (Fig.1c, d Orange bars are KD, white CD). Also, BHB is significantly higher in the fasting phase in KD-fed females than in males, *p* < 0.05 (Fig. 1d, KD female vs KD male).

Microglia are intimately involved with synaptic plasticity, and microglial activation/inflammation has been proposed as a potential mechanism in Alzheimers^44^. We observe that KD significantly reduces Iba-1 and CD11b, markers of microglial activation, but had no significant effect on markers of Astrocyte activation (Fig. 4). Thus it is possible that KD’s synaptic plasticity mechanism of LTP rescue may involve reduced age- and Aβ induced microglial inflammation.

LTP decline is the earliest neurophysiologically-measurable feature in both APP/PS1 mice^24^ and in human AD^38,39^, other deficits occur later. While in locomotor tests Open Field and Y-maze distance, significant effects of diet were observed (Supl. Fig. 4a, b), in memory tests, Y-maze Alternation, Barnes Maze latency, Barnes Maze % time in quadrant effects were much more modest (Supl. Fig. 5a–c).

We interpret the data in the following way. In both the APP/PS1 mouse model of AD and in humans, LTP is the earliest neurophysiologically measurable parameter/biomarker of AD to fall. In APP/PS1 mice, hippocampal LTP is normal at 6 months but declines significantly at 8 months^24,45^, coincident with the maximal Aβ deposition at 8 months^46^, but before the maximal expression of neuroinflammation and neurobehavioral pathophysiology at 12 months and later.

Similarly, in humans, as in APP/PS1 mice, LTP-like activity deterioration is the first and earliest biomarker of AD progression^39,46^. The most favored current method to measure LTP-like activity in humans is transcranial magnetic stimulation (TMS)^39^. In a recent study using TMS to measure LTP-like activity at a mean age of 65 years, LTP-like activity of 130 MEP amplitude in healthy subjects progressed to a significant reduction in LTP-like activity of 95 MEP in individuals diagnosed with Mild Cognitive Impairment (MCI), that progressed to ~80MEP in individuals with either prodromal AD or fully manifested AD^38^. The interpretation was that a drop in LTP-like activity is the earliest biomarker of AD progression in humans. This deficit occurs maximally in the normal-to-MCI phase, whereas other non-LTP pathophysiological processes cause deficits later in AD’s progression, i.e., the ‘Prodromal AD’ and ‘Manifest AD’ phases^38^. So in APP/PS1 mice and apparently in humans, there is a significant decline in LTP-like activity at the earliest stages of disease, preceding or coincident with MCI in the human condition. At 6 months wild type C57BL/6 and APP/PS1 mice have fEPSP signals of ~150 fEPSP, KD vs CD treatment of APP/PS1 mice rescued their LTP deficit at 13 months from ~110 fEPSP expected to ~160 fEPSP, not significantly different from WT mice. In humans of age 65 healthy individuals had LTP-like activity of 130 MEP, whereas those diagnosed with MCI had a significant fall to 95 MEP. Since the KD is currently consumed by humans and is used therapeutically for the neurological condition of epilepsy, and the magnitude of LTP-like fall is similar at the earliest stages of disease in APP/PS1 mice (36%) as in human MCI (37%), one could imagine a clinical translation of these findings. In addition, there are multiple Ketone supplements on the market whose consumption significantly increases systemic BHB exposure.

We suggest that KD and BHB may work through the following 3 mechanisms: (1) increased synaptic plasticity, (2) increased synaptic support through biochemical pathways p-ERK, p-CREB, BDNF, and (3) reduced microglial inflammation. We suggest these mechanisms are most relevant during the ‘MCI phase’ which occurs from month 8 to 10 in APP/PS1 mice, and the ~6 y of MCI preceding prodromal and Manifest AD in humans^38^. We suggest that KD, Ketone supplements, and BHB formulation are most likely to have an impact on this earliest MCI phase of human AD, and could potentially extend/rescue this phase in humans, as KD/BHB appears to rescue MCI in APP/PS1 mice. Furthermore, since KD is already consumed by humans, and since multiple ketone supplements are on-market and available, rapid translation to human clinical trials of efficacy for delay of this MCI inital phase of AD could occur.

## Methods

### Mice and diets

All protocols involving mouse models were approved by the Institutional Animal Care and Use Committee of the University of California Davis. C57BL/6 and APP/PS1 [B6.Cg-Tg(APPswe,PSEN1dE9)85Dbo/Mmjax] mice were originally purchased from the Jackson laboratory. The mice were housed in HEPA-filtered rooms with controlled temperature (22–24 °C) and humidity (40-60%). The mice were maintained on 12 h light and dark cycle and allowed free choice consumption of water throughout the study. Both male and female mice were used for this study. The mice were bred in-house. Following weaning, the mice were group-housed (up to 4 mice per cage) and provided ad libitum access to a chow diet (TestDiet 5001; TestDiet, St. Louis, MO). At six months of age, the mice were counter-balanced by body weight, assigned to diet groups (control or ketogenic diets), and singly housed for the duration of the study to allow accurate control of food intake. The ketogenic diet contained (%kcal) <0.5% carbohydrates, 10% protein, and 90% fat, and the control diet contained 74% carbohydrates, 10% protein, and 16% fat. The compositions of the diets are published^47^. The mice were fed isocaloric (11.2 kcal/d) amounts of the diets. This diet amount was selected because we have fed male^16^ and female^47^ C57BL/6 mice this amount in previous studies, and this level of intake has maintained middle-aged body weight with mice housed in the same facility and under the same husbandry conditions. We have complied with all relevant ethical regulations for animal use.

### Body composition, β-hydroxybutyrate measurement, and tissue collection

Body composition was measured in the mice at 13 months of age using NMR relaxometry (EchoMRI-100H, EchoMRI LLC, Houston, TX). Prior to termination, blood β-hydroxybutyrate (BHB) was measured in the mice using a Precision Xtra glucose and ketone monitoring system (Abbott Diabetes Care Inc., Alameda, CA) with a tail nick at 3-h postprandial and after a 12-h overnight fast. Following the morning BHB measurement, the mice were euthanized by exsanguination under isoflurane anesthesia, and tissues were collected for gene expression measurements. Tissues were snap-frozen in liquid nitrogen.

### Induction of hippocampal long-term potentiation (LTP) by high-frequency stimulation

The preparation of mouse hippocampal slices and the induction of LTP by high-frequency stimulation of the Schaffer collateral afferents were conducted on a subset of mice as we previously described^48^. Briefly, coronal slices (300 µm) of mouse hippocampus were prepared from WT and APP/PS1. The animals were subjected to deep anesthesia with isoflurane and decapitated. The brain was rapidly removed and transferred to a modified artificial cerebrospinal fluid (HI-ACSF) containing (in mM): 220 sucrose, 2 KCl, 0.2 CaCl_2,_ 6 MgSO_2_, 26 NaHCO_3_, 1.3 NaH_2_PO_4_, and 10 d-glucose (pH 7.4, set by aeration with 95% O_2_ and 5% CO_2_). Coronal brain slices were cut in ice-cold modified artificial cerebrospinal fluid (ACSF) with the use of a DTK-1000 D.S.K. Microslicer (TedPella, Inc., Redding, CA, USA). The slices were immediately transferred into an ACSF solution containing (in mM): 126 NaCl, 3 KCl, 2 CaCl_2_, 1 MgCl_2_, 26 NaHCO_3_, 1.25 NaH_2_PO_4_, and 10 d-glucose (pH 7.4, set by aeration with 95% O_2_ and 5% CO_2_) for at least 40 min at controlled temperature of 37 °C. After subsequent incubation for at least 1 h at room temperature, hemi-slices were transferred to the recording chamber, which was perfused with standard ACSF at a constant flow rate of ~2 ml/min. Recordings of field excitatory postsynaptic potentials (fEPSPs) were obtained from the stratum radiatum of the CA1 region of the hippocampus after stimulation of the Schaffer collateral afferents. For BHB experiments, hemi-slices from 15-month-old APP/PS1 mice were incubated in 3 mM BHB for 1 h prior to recording. Extracellular recording electrodes were prepared from borosilicate capillaries with an outer diameter of 1.5 mm (Sutter Instruments) and were filled with 3 M NaCl (resistance, 1–2 MΩ). The baseline stimulation rate was 0.05 Hz. fEPSPs were filtered at 2 kHz and digitized at 10 kHz with a Multiclamp 700B amplifier (Molecular Devices, Sunnyvale, CA). Data were collected and analyzed with pClamp 10.3 software (Molecular Devices). Slope values of fEPSPs were considered for quantitation of the responses. After 10 min of stable baseline recording of fEPSPs evoked every 20 s by application of a constant current pulse of 0.2–0.4 mA with a duration of 60 ms at the current intensity set to evoke 50–60% of the maximal response, LTP was elicited by high-frequency stimulation (HFS), consisting of two trains of 100-Hz (1 s) stimulation with the same intensity and pulse duration used in sampling of baseline fEPSPs. The recording was then continued for 60 min with stimulation of fEPSPs every 20 s.

### RNAseq and gene ontology by ingenuity pathway analysis

RNA was isolated starting from 10 mg of frozen murine brain cortex (CD group *n* = 4, KD group *n* = 3), using the RNeasy Mini Kit (QIAGEN, Hilden, Germany) and following the manufacturer’s instructions. Extracted RNA was used for NGS library preparation following Illumina instruction (TruSeq Stranded Total RNA Library Prep Gold), while sequencing was performed on NovaSeq^TM^ 6000 (Illumina) running in a 50-bp pair-end mode.

Quality-control checks on raw sequences were performed using FASTQC (Babraham Institute). Samples were then aligned on the murine reference genome (*Mus Musculus* UCSC, mm10), using STAR. Mapped sequences were processed with HTSeq software implemented into STAR to count reads *per* gene as a first raw measure of gene expression levels. TMM (Trimmed Mean of *M*-values) normalization and differential gene expression analysis were performed with the R package *edgeR*. All differentially expressed genes (DEGs) with raw *p*-value ≤ 0.01 were used as input for Ingenuity Pathway Analysis (IPA) (QIAGEN Redwood City) in order to identify pathways to which DEGs belong. All RNA-seq results are plotted using ggplot2 R package.

### Tissue homogenate preparation and Western blot analysis

Brain tissues were homogenized in lysis buffer (150 mM NaCl, 10 mM NaH_2_PO_4_, 1 mM EDTA, 1% Triton X-100, 0.5% SDS) with protease inhibitor cocktail and phosphatase inhibitor (Sigma). Equivalent amounts of protein were analyzed by 4–20% Tris-Glycine gel electrophoresis (Invitrogen). Proteins were transferred to polyvinylidene difluoride membranes and probed with antibodies. Visualization was enabled using enhanced chemiluminescence (GE Healthcare Pharmacia). The following primary antibodies (dilutions) were used: anti-phosphoCREB (1:1000, Cell Signaling, #9198), anti-CREB (1:1000, Cell Signaling, #9197), anti-phosphoERK (1:1000, Cell Signaling, #9106), anti-ERK (1:1000, Cell Signaling, #9102), anti-phospho-CaMKII (1:1000, Cell Signaling, #12716), anti-CaMKII (1:1000, Cell Signaling, #4436), anti-PSD95 (1:1000, Cell Signaling, #36233), anti-BDNF (1:1000, Sigma, #AB1534SP), Iba-1 (1:1000, Santa Cruz Biotechnology, sc-32725), CD68 (1:1000, Bio-Rad, MCA341GA), Dectin-1 (1:1000, Invitrogen, PA5-34382), CD11b (1:1000, Bio-Rad, MCA711), and β-actin (1:5000, Cell Signaling, #3700). Secondary antibodies were HRP-conjugated anti-rabbit or anti-mouse antibodies (1:1000, Cell Signaling, #7074 and #7076).

### ELISA quantification

For ELISA quantification of total BDNF, tissue samples were homogenized in lysis buffer (100 mM TRIS, pH 7.4; 150 mM NaCl; 1 mM EGTA; 1 mM EDTA; 1% Triton X-100; 0.5% Sodium deoxycholate; proteinase inhibitor mix), and centrifuged for 20 min at 15,000 rpm at 4 °C. The supernatants were directly used for the total BDNF. Concentrations of BDNF were measured using the Quantikine sandwich ELISA kit (R&D systems, Minneapolis, MN).

### Tissue homogenate preparation and Aβ ELISA

Brain tissue samples from APP/PS1 treated with CD or KD were fractionated into Tris-buffered saline (TBS)-soluble and TBS-insoluble, SDS-soluble fractions, which were used for Aβ42 quantification by Human Aβ42 ELISA kit (Invitrogen). Briefly, brain tissues were homogenized in TBS with protease inhibitors, followed by centrifugation (100,000*g*, 1 h, at 4 °C). The supernatants were collected as the TBS-soluble fraction. The pellets were homogenized in 2% SDS with protease inhibitors, followed by centrifugation (100,000*g*, 1 h, at 4 °C). The supernatants were collected as the TBS-insoluble, SDS-soluble fraction.

### Immunofluorescence staining

Frozen mouse sections (16 µm) were fixed with 4% PFA and blocked with 10% normal goat serum with 0.3% Triton-X. Fixed brain sections were incubated with primary antibody, anti-GFAP-Alexa Flour®488 (1:400, abcam, ab302977) or anti-Iba1 (1:400, Biocare Medical, #290), overnight at 4 °C. For Iba1, sections were then incubated with Alexa Fluor-conjugated secondary antibody (1:700, Invitrogen, #A32740). For the detection of amyloid plaques, 400 nmol/L FSB (Sigma) was used. Immunostained slides were imaged under a Nikon Eclipse E600 microscope and photographed by a digital camera (SPOT RTsCMOS, SPOT Diagnostics). The images were randomly taken in a blind fashion within a defined anatomic region. The images were transformed to 8-bit grayscale and analyzed by the ImageJ program.

### Mouse neurobehavioral measurements

Mouse neurobehavioral measurements were conducted as we previously described^49^.

#### Open field

Mice were placed in a 40 × 40 × 40 cm white acrylic box and allowed to explore the arena for 15 min. The animal movement was recorded using an overhead camera. The videos were analyzed using Ethovision XT16 software (Noldus, Wageningen, the Netherlands) to determine the total distance traveled.

#### Y-maze

Mice were placed in a white acrylic Y-shaped maze for this test. Each arm of the maze was 120° from each other and 35 × 8 × 15 cm (L × H × W) in dimension. Trials were recorded using an overhead camera, and videos were analyzed using Ethovision XT16 software (Noldus, Wageningen, the Netherlands). An arm entry was counted when the center point of the mouse traveled >4 cm into an arm. A non-repeating triplet is defined as when the mouse enters three different arms consecutively. The percent alternation was calculated as ((number of non-repeating triplets) ÷ (number of total arm entries − 2)) × 100%.

#### Barnes maze

The Barnes maze consisted of a white acrylic 92 cm plastic circular disk with twenty 5 cm holes evenly distributed on the periphery, and an overhead LED light source was used to illuminate the maze elevated ~75 cm above the ground. Signs were placed around the maze to be used as visual cues. A black escape box equipped with a step and fresh bedding was secured under the target hole as a shelter for the mice. During the training trials, mice were placed in the middle of the maze under an inverted opaque bucket. After 10 s, the light was turned on and the bucket was immediately lifted. Mice were allowed to explore the maze for 3 min or until they entered the escape box through the target hole. If the mice did not enter the target hole within 3 min, the mice were directed to the target hole, where they were allowed to rest for at least 30 s. Mice were trained for 3 trials a day for 3 days and the intertrial interval was 15–20 min. On the 4th day, probe day, the escape box was removed, and the mice were allowed to explore for 2 min. Mice that did not find the target hole in 2 min in the probe trial were considered outliers and were excluded from the analysis. Videos were recorded and videos analyzed using the Ethovision XT16 software (Noldus, Wageningen, the Netherlands). Latency to the target hole and percentage time spent in the target quadrant (quarter of the maze with the target hole in the middle) were calculated automatically using the software.

### Statistics and reproducibility

Statistical analysis was performed using GraphPad Prism 10 software. All data are presented as means ± standard error of mean^50^ or median ± interquartile range. Group comparisons were performed using unpaired Student’s two-sample *t*-test, one-way analysis of analysis (ANOVA), non-parametric Kruskal–Wallis test, or two-way ANOVA with Tukey’s post hoc tests to control a family-wise type I error at 5% for multiple testing for pairwise comparisons as appropriate. The analysis of residuals and Bartlett’s test were performed to validate underlying assumptions of parametric tests prior to statistical inference. For the behavioral tests, observations were considered extreme outliers if values were greater than three standard deviations from the mean according to the three-sigma rule, and those outliers were excluded from further analyses. Prior to statistical inference tests, the analysis of residuals was performed to validate the normality and homoscedasticity assumptions using QQ and residual plots as well as D’Agostino & Pearson test for normality. Where appropriate, log transformation was applied to data to approximately conform to normality and homogeneity of variances prior to statistical inference tests. Outliers within each group were identified using the ROUT method, a robust nonlinear regression-based method^51^, and removed from statistical analysis. For behavior tests, two-way ANOVA was used to evaluate differences between genotypes and diets, followed by Tukey’s post hoc test for pairwise group comparisons. Unpaired Students’ two-sample *t*-test was used to compare differences between diet groups within a sex as appropriate. Exact sample sizes and statistical tests used for each comparison were provided in corresponding figure legends. Two-tailed *p*-values < 0.05 were considered to be statistically significant. In support of reproducibility, at least 3 replicates of each experiment were done.

### Reporting summary

Further information on research design is available in the Nature Portfolio Reporting Summary linked to this article.

### Supplementary information

Supplementary Information

Description of Additional Supplementary Files

Supplementary Data

Reporting Summary

## Data Availability

The source data behind the graphs in the paper can be found in the Excel file named Supplementary Data. Uncropped blots are provided in Supplementary Fig. 6. RNAseq data are deposited on the GEO repository and are accessible with the number GSE230469. Other data are available upon request after acceptance.
