# Supplementary material for: Ketogenic diet and BHB rescue the fall of long-term potentiation in an Alzheimer’s mouse model and stimulates synaptic plasticity pathway enzymes
Source: Commun Biol. 2024 Feb 16;7:195. doi: 10.1038/s42003-024-05860-z (PMC10873348; doi:10.1038/s42003-024-05860-z)
Supplement: Supplementary file 1 — Supplementary Information [file 42003_2024_5860_MOESM1_ESM.pdf]

Supplementary Figure 1

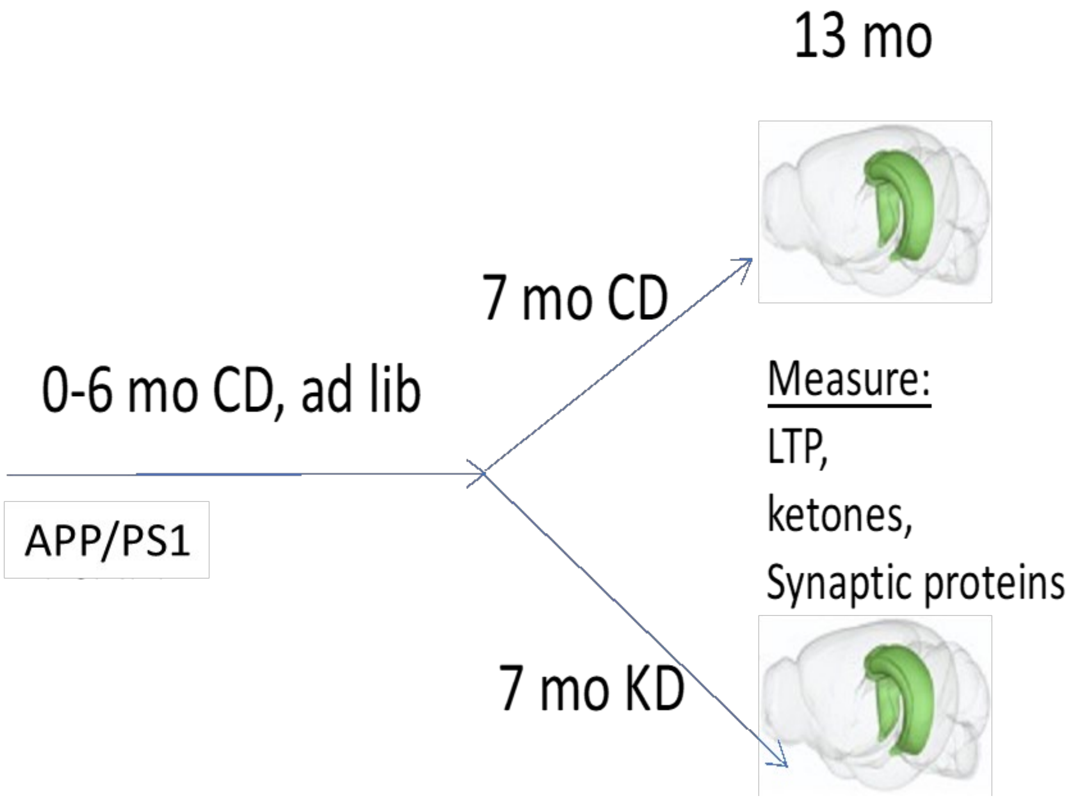

Supplementary Figure 1: Overall study design. 6 months old APP/PS1 were divided into two groups, one received a standard carbohydrate-rich diet (CD) for 7 months, while the other group received a ketogenic diet (KD). A 12-13 months of age mice were used for neurobehavior testing, LTP, ketones and biochemical measurements.

Supplementary Figure 2

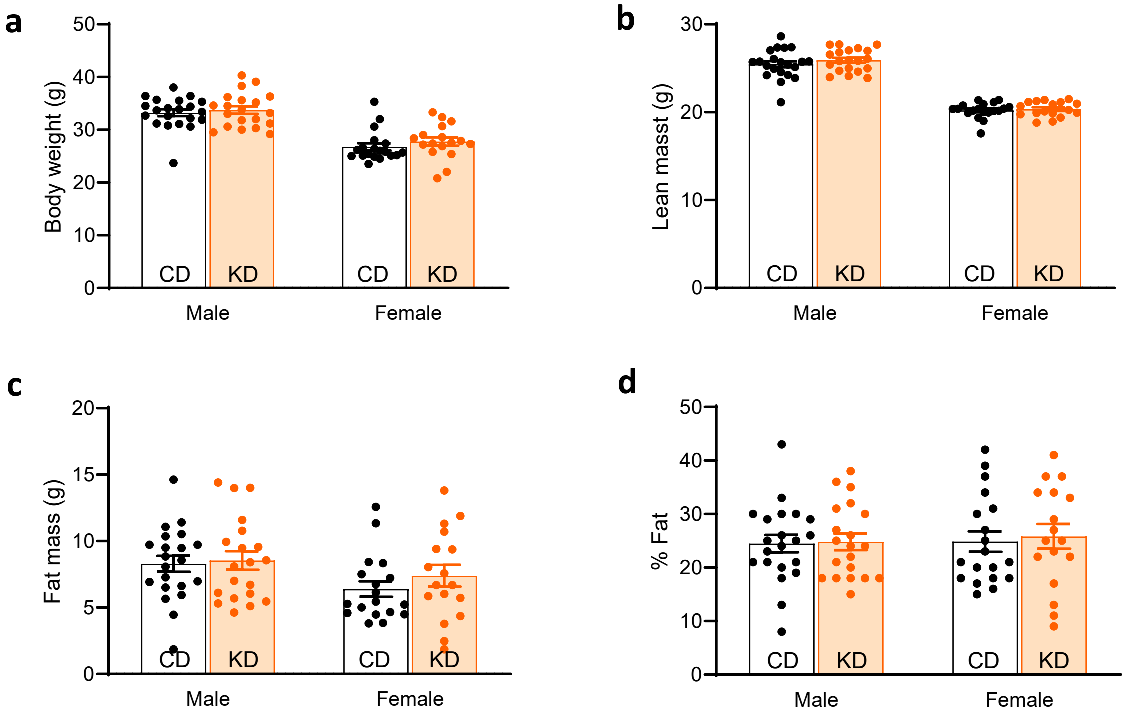

Supplementary Figure 2: Ketogenic diet does not alter body weight or composition in male or female APP/PS1 mice when fed in isocaloric amounts to a control diet. **a.** Body weight, **b.** Lean Mass, **c.** Fat Mass, and **d.** Percent Body Fat measured at 13-months of age did not significantly differ between male mice fed the control (CD) or ketogenic (KD) and female mice fed the CD or KD. (n=18-21). Data are presented as mean ± SEM.

Supplementary Figure 3

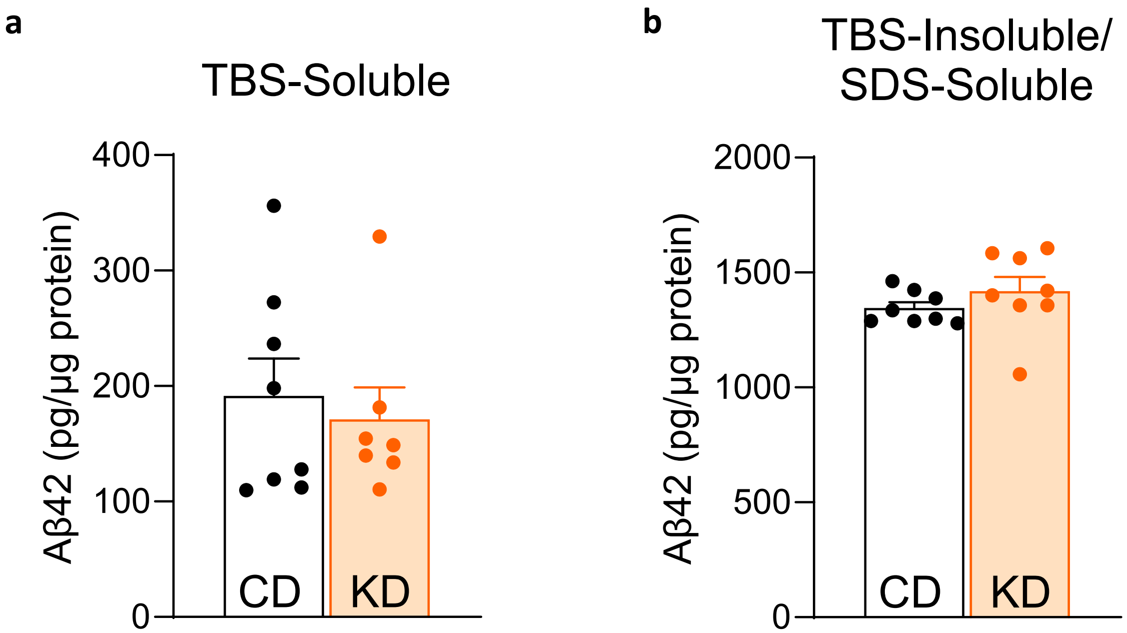

Supplementary Figure 3: Ketogenic diet does not reduce Aβ. **a-b.** The fresh brains were fractionated into TBS-soluble and TBS-insoluble, SDS-soluble fractions, which were used for ELISA quantification of Aβ42 (n=4 male and 4 female). Data are presented as mean ± SEM

Supplementary Figure 4

a

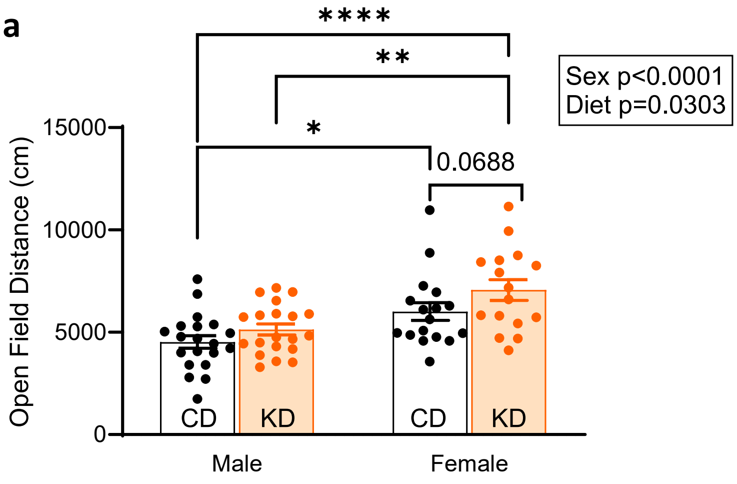

b

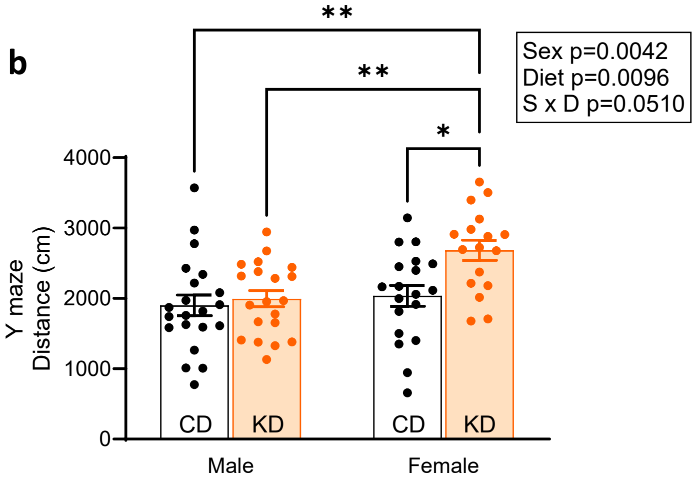

Supplemental Figure 4: a. Ketogenic diet increases distance traveled in an open field in APP/PS1 mice. Open field total distance covered was significantly increased in females and mice on a ketogenic diet (n=16-20). Two-way ANOVA tests show significant differences between sexes ($F(1, 69) = 20.53$, $p<0.0001$) and diet ($F(1, 69) = 4.893$, $p=0.0303$). **b.** Ketogenic diet increases APP/PS1 mouse exploration in Y-maze. Both female mice and mice fed a ketogenic diet showed significant increase in distance traveled during y-maze (n=17-21). Two-way ANOVA tests show significant differences between sexes ($F(1, 73) = 8.754$, $p=0.0042$), diets, ($F(1, 73) = 7.065$. $p=0.0096$) as well as an interaction between sex and diet ($F(1, 73) = 3.935$, $p=0.0510$).Data are presented as mean ± SEM and include individual data points. CD= Control diet, KD= Ketogenic Diet *p<0.05, **p<0.01, ****p<0.0001

Supplementary Figure 5

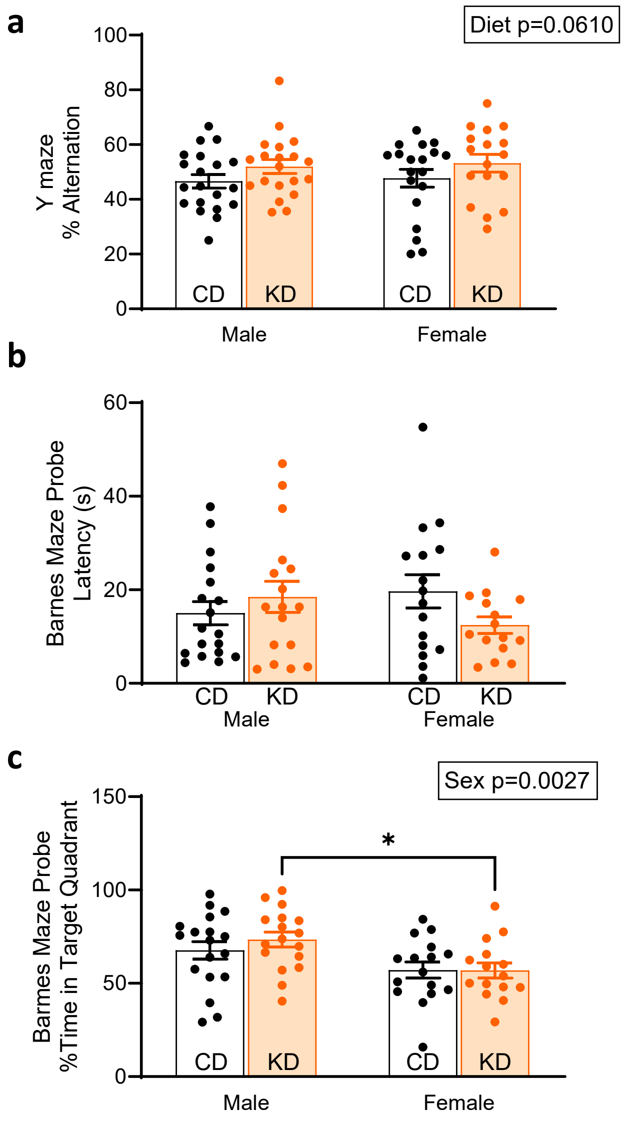

Supplementary Figure 5: Trend for improved working memory in ketogenic diet fed APP/PS1 mice. **a.** Y-maze % alternating triplets was determined in control (CD) and ketogenic (KD) diet fed APP/PS1 mice (n=17-20). Two-way ANOVA tests showed a trend for differences between diet groups ($F(1, 72) = 3.623$, $p=0.0610$). Spatial learning and memory, assessed as **b.** latency to target and **c.** % time in target quadrant in the Barnes maze test, was determined in CD and KD fed APP/PS1 mice. Two-way ANOVA tests showed a sex effect in % time in target quadrant $F(1, 62) = 9.739$, $p=0.0027$. Data are presented as mean ± SEM and include individual data points.

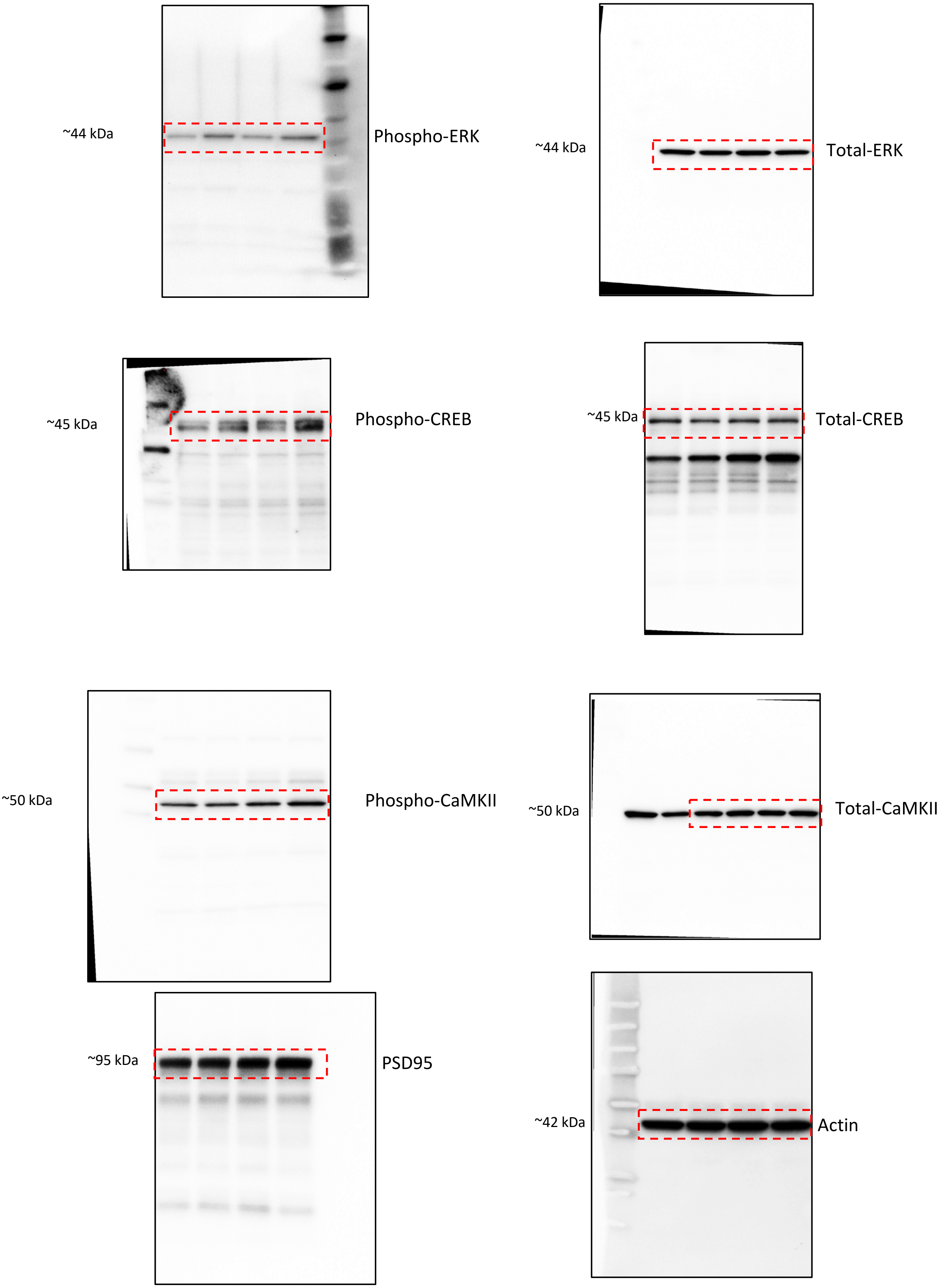
~44 kDa
Phospho-ERK
~44 kDa
Total-ERK
~45 kDa
Phospho-CREB
~45 kDa
Total-CREB
~50 kDa
Phospho-CaMKII
~50 kDa
Total-CaMKII
~95 kDa
PSD95
~42 kDa
Actin

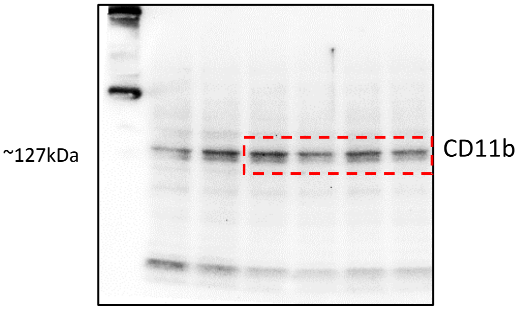
~127kDa
CD11b

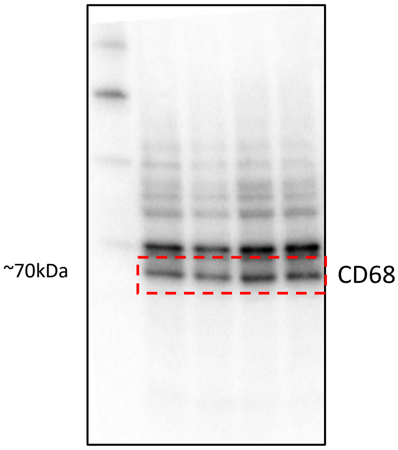
~70kDa
CD68

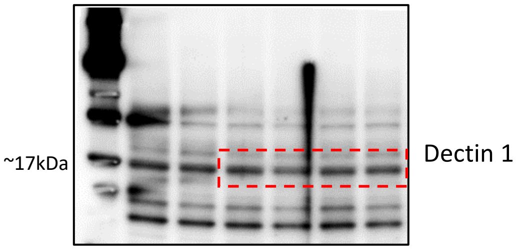
~17kDa
Dectin 1

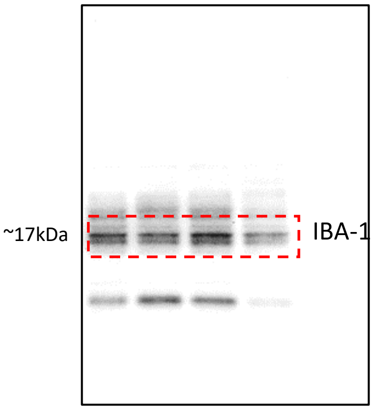
~17kDa
IBA-1

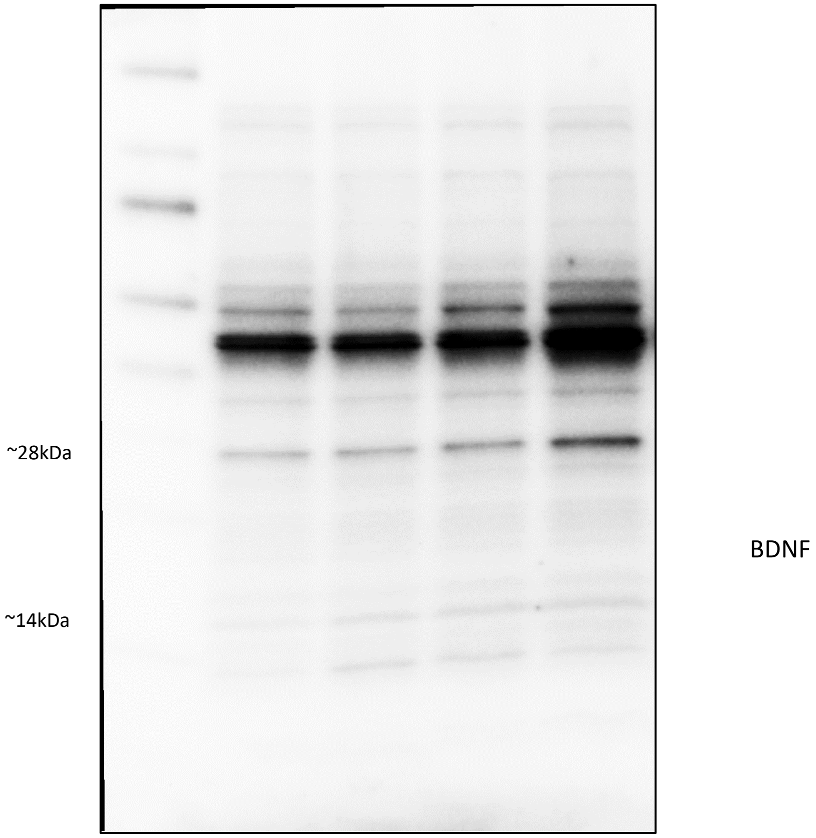

Supplementary Figure 6: Uncropped gel/blot
