# Supplementary material for: Ketogenic diet and BHB rescue the fall of long-term potentiation in an Alzheimer’s mouse model and stimulates synaptic plasticity pathway enzymes
Source: Commun Biol. 2024 Feb 16;7:195. doi: 10.1038/s42003-024-05860-z (PMC10873348; doi:10.1038/s42003-024-05860-z)
Supplement: Supplementary file 4 — Reporting Summary [file 42003_2024_5860_MOESM4_ESM.pdf]

nature portfolio

Corresponding author(s): Cortopassi, Gino & Maezawa, Izumi

Last updated by author(s): Jan 12, 2024

Reporting Summary

Nature Portfolio wishes to improve the reproducibility of the work that we publish. This form provides structure for consistency and transparency in reporting. For further information on Nature Portfolio policies, see our Editorial Policies and the Editorial Policy Checklist.

Statistics

For all statistical analyses, confirm that the following items are present in the figure legend, table legend, main text, or Methods section.

n/a	Confirmed	
☐	☒	The exact sample size (n) for each experimental group/condition, given as a discrete number and unit of measurement
☐	☒	A statement on whether measurements were taken from distinct samples or whether the same sample was measured repeatedly
☐	☒	The statistical test(s) used AND whether they are one- or two-sided<br>*Only common tests should be described solely by name; describe more complex techniques in the Methods section.*
☐	☒	A description of all covariates tested
☐	☒	A description of any assumptions or corrections, such as tests of normality and adjustment for multiple comparisons
☐	☒	A full description of the statistical parameters including central tendency (e.g. means) or other basic estimates (e.g. regression coefficient) AND variation (e.g. standard deviation) or associated estimates of uncertainty (e.g. confidence intervals)
☐	☒	For null hypothesis testing, the test statistic (e.g. F, t, r) with confidence intervals, effect sizes, degrees of freedom and P value noted<br>*Give P values as exact values whenever suitable.*
☒	☐	For Bayesian analysis, information on the choice of priors and Markov chain Monte Carlo settings
☒	☐	For hierarchical and complex designs, identification of the appropriate level for tests and full reporting of outcomes
☐	☒	Estimates of effect sizes (e.g. Cohen's d, Pearson's r), indicating how they were calculated

Our web collection on statistics for biologists contains articles on many of the points above.

Software and code

Policy information about availability of computer code

Data collection	We used Graphpad Prism v. 9.5.1 to statistically analyze and graph data.<br>Also, Molecular Device pClamp v10.3, Bio-Rad Image Lab v5.1, Molecular Device SoftMax Pro v7.1
Data analysis	We used Graphpad Prism v. 9.5.1 and R to statistically analyze and graph data.

For manuscripts utilizing custom algorithms or software that are central to the research but not yet described in published literature, software must be made available to editors and reviewers. We strongly encourage code deposition in a community repository (e.g. GitHub). See the Nature Portfolio guidelines for submitting code & software for further information.

Data

Policy information about availability of data

All manuscripts must include a data availability statement. This statement should provide the following information, where applicable:

- Accession codes, unique identifiers, or web links for publicly available datasets
- A description of any restrictions on data availability
- For clinical datasets or third party data, please ensure that the statement adheres to our policy

Data will be provided upon request.

Research involving human participants, their data, or biological material

Policy information about studies with human participants or human data. See also policy information about sex, gender (identity/presentation), and sexual orientation and race, ethnicity and racism.

Reporting on sex and gender	No human data.
Reporting on race, ethnicity, or other socially relevant groupings	*Please specify the socially constructed or socially relevant categorization variable(s) used in your manuscript and explain why they were used. Please note that such variables should not be used as proxies for other socially constructed/relevant variables (for example, race or ethnicity should not be used as a proxy for socioeconomic status).*<br>*Provide clear definitions of the relevant terms used, how they were provided (by the participants/respondents, the researchers, or third parties), and the method(s) used to classify people into the different categories (e.g. self-report, census or administrative data, social media data, etc.)*<br>*Please provide details about how you controlled for confounding variables in your analyses.*
Population characteristics	*Describe the covariate-relevant population characteristics of the human research participants (e.g. age, genotypic information, past and current diagnosis and treatment categories). If you filled out the behavioural & social sciences study design questions and have nothing to add here, write "See above."*
Recruitment	*Describe how participants were recruited. Outline any potential self-selection bias or other biases that may be present and how these are likely to impact results.*
Ethics oversight	*Identify the organization(s) that approved the study protocol.*

Note that full information on the approval of the study protocol must also be provided in the manuscript.

Field-specific reporting

Please select the one below that is the best fit for your research. If you are not sure, read the appropriate sections before making your selection.

☒ Life sciences ☐ Behavioural & social sciences ☐ Ecological, evolutionary & environmental sciences

For a reference copy of the document with all sections, see nature.com/documents/nr-reporting-summary-flat.pdf

Life sciences study design

All studies must disclose on these points even when the disclosure is negative.

Sample size	Sample sizes were determined based on our previous experiments in LTP sample size required in the PS1/APP mice, and biochemical measurements of synaptic plasticity enzymes.
Data exclusions	No data were excluded.
Replication	We didn't replicate our experiment.
Randomization	Animals are randomly divided into groups by sex.
Blinding	Experimenter not blinded to mouse identity, for neurophysiological experiments (LTP).

Reporting for specific materials, systems and methods

We require information from authors about some types of materials, experimental systems and methods used in many studies. Here, indicate whether each material, system or method listed is relevant to your study. If you are not sure if a list item applies to your research, read the appropriate section before selecting a response.

Materials & experimental systems

n/a	Involved in the study	
☐	☒	Antibodies
☒	☐	Eukaryotic cell lines
☒	☐	Palaeontology and archaeology
☐	☒	Animals and other organisms
☒	☐	Clinical data
☒	☐	Dual use research of concern
☒	☐	Plants

Methods

n/a	Involved in the study	
☒	☐	ChIP-seq
☒	☐	Flow cytometry
☒	☐	MRI-based neuroimaging

Antibodies

Antibodies used	The following primary antibodies (dilutions) were used: anti-phosphoCREB (1:1000, Cell Signaling), anti-CREB (1:1000, Cell Signaling), anti-phosphoERK (1:1000, Cell Signaling), anti-ERK (1:1000, Cell Signaling), anti-phospho-CaMKII (1:1000, Cell Signaling), anti-CaMKII (1:1000, Cell Signaling), anti-PSD95 (1:1000, Cell Signaling), anti-BDNF (1:1000, Sigma) and β-actin (1:5000, Cell Signaling). Secondary antibodies were HRP-conjugated anti-rabbit or anti-mouse antibody (1:1000, Cell Signaling), Iba-1 (1:1000, Santa Cruz Biotechnology, sc-32725), CD68 (1:1000, Bio-Rad, MCA341GA), Dectin-1 (1:1000, Invitrogen, PA5-34382), CD11b (1:1000, Bio-Rad, MCA711)
Validation	Each primary antibody was selected and validated based on several criteria (publication record, company QC information, compatibility with experimental material being probed and other antibodies used in tandem). Once received, each antibody was optimized for primary and secondary dilution. Antibodies recognizing multiple bands were further optimized or alternatives chosen.

Animals and other research organisms

Policy information about studies involving animals; ARRIVE guidelines recommended for reporting animal research, and Sex and Gender in Research

Laboratory animals	All protocols involving mouse models were approved by the Institutional Animal Care and Use Committee of the University of California Davis. C57BL/6 and APP/PS1 [B6.Cg-Tg(APPswe,PSEN1dE9)85Dbo/Mmjax] mice were originally purchased from the Jackson laboratory
Wild animals	None
Reporting on sex	Equal numbers of Males and Females were used in the study design, sex-specific effects were observed for Ketogenic Diet's effect on BDNF.
Field-collected samples	None
Ethics oversight	All work was done in IACUC-approved sites under active animal use protocols.The active IACUC-approved protocol, 'Ketogenic diet, ketone supplements and aging', 22859, was used

Note that full information on the approval of the study protocol must also be provided in the manuscript.
